# The Anti-Inflammatory Properties of the Topical Application of Human Milk in Dermal and Optical Diseases

**DOI:** 10.1155/2020/4578153

**Published:** 2020-07-23

**Authors:** Leila Amiri-Farahani, Zahra Sharifi-Heris, Faraz Mojab

**Affiliations:** ^1^Department of Reproductive Health and Midwifery, Nursing Care Research Center, School of Nursing and Midwifery, Iran University of Medical Sciences, Tehran, Iran; ^2^Sue & Bill Gross School of Nursing, University of California at Irvine, Irvine, CA, USA; ^3^Department of Pharmacognosy, School of Pharmacy, Shahid Beheshti University of Medical Sciences, Tehran, Iran

## Abstract

**Methods:**

The various datasets including Ovid, PubMed, Google Scholar, Science Direct, Magiran, Irandoc, SID, and IranMedex were searched from 1990 to 2018. From the 119 reviewed articles, 20 articles were selected to be included in the current article.

**Results:**

There is a consensus among the literature and ancient texts regarding the effectiveness of THM in curing the various types of skin damages, such as cord separation, atopic dermatitis, diaper dermatitis, conjunctivitis, scratches, insect bite, perineal ulcer, and nipple ulcer. However, the importance of its application has not been given much attention.

**Conclusion:**

According to the information obtained from the articles reviewed, the THM appears to be an effective, safe, and available treatment compared to conventional chemical treatments. This study suggests THM as an alternative remedy to minimize the frequent use of chemical-based treatments. More research may be beneficial to reach certainty in terms of curative properties of THM in similar or different injuries in different populations.

## 1. Background

The human milk (HM) consumption, orally or topically, has beneficial effects on both mother and child's health. According to the literature, it was shown that feeding with HM leads to increased resistance to asthma, diarrhea, respiratory infections, urinary tract infections, otitis media, intestinal infections, celiac disease, breast cancer, leukemia, neonatal diabetes, eczema, allergic diseases, influenza, cardiovascular disease, and mental disorders and reduced risk of obesity in childhood and adulthood [1]. The advantages of breastfeeding for mothers include postpartum weight loss, lower risk for breast, ovarian, and endometrial cancers, bone strength, and lower risk of joint rheumatoid arthritis [1, 2].

Today, the topical application of human milk (THM) has been documented to treat the optical inflammations including conjunctivitis in ancient Egypt, Greek, India, and Rome for decades [3]. The THM also has been used as a traditional practice in healing the infants' umbilical cords and accelerating the cord separation in the communities in Kwazulu-Natal, as well as in some parts of Kenya, Turkey, India, and China [4]. Furthermore, eczema and diaper dermatitis as common skin inflammations among infants have been cured using THM [5]. The results of a qualitative study in Zambia show the positive experiences following the THM application to prevent and treat the umbilical cord inflammations [6]. The promising effects of THM also have been indicated among women in improving the nipple sore and perineal ulcer [7, 8].

HM consists of anti-inflammatory and antioxidant components, including vitamins A, C, and E, catalase enzymes, glutathione peroxidases, prostaglandins, and platelet activators as well as leukocytes that regulate the immune system [9, 10]. The aforementioned components may be the potential underlying cause for the curative properties of the THM.

Today, the tendency toward traditional treatments is increasing across the world especially developed countries [11]. A variety of studies have shown the negative consequences of conventional medicine in various populations including children. According to an investigation, people reported expensiveness, side effects, the difference in physicians' opinions, and distrust in physicians as potential disadvantages of conventional medications [12]. The THM seems to be a safe, accessible, trustable, and noninvasive alternative for domestic application, especially in mothers and infants, as it is produced in the human body and contains natural anti-inflammatory components [13].

Despite the effectiveness of THM in different cultures and ethnicities in underdeveloped and developing countries, its use has been less widely considered among health professionals and staff especially in developed countries [5]. The current study aimed to review the literature to assess the effect of THM on various inflammations.

## 2. Methods

### 2.1. Design

The current literature review study was completed following the academic standards for conducting integrative literature reviews [14]. As an additional element of quality assurance, we applied the Preferred Reporting Items for Systematic Reviews and Meta-Analyses (PRISMA) to structure the study [15]. The studies were included if they were interventional or review papers.

### 2.2. Setting

Journal articles were examined in PubMed, CINAHL, PsycINFO, Web of Science, Ovid, Google Scholar, Science Direct, Cochrane Library, Magiran, Irandoc, and SID, using keywords that probe the significance of HM applied topically as a form of treatment for ulcers and inflammatory lesions: “human milk,” “colostrum,” “topical application,” “wound,” “wound healing,” “ulcer,” and “sore.” These articles were peer-reviewed and published from 1990 to 2018.

### 2.3. Sample

**Figure 1:**
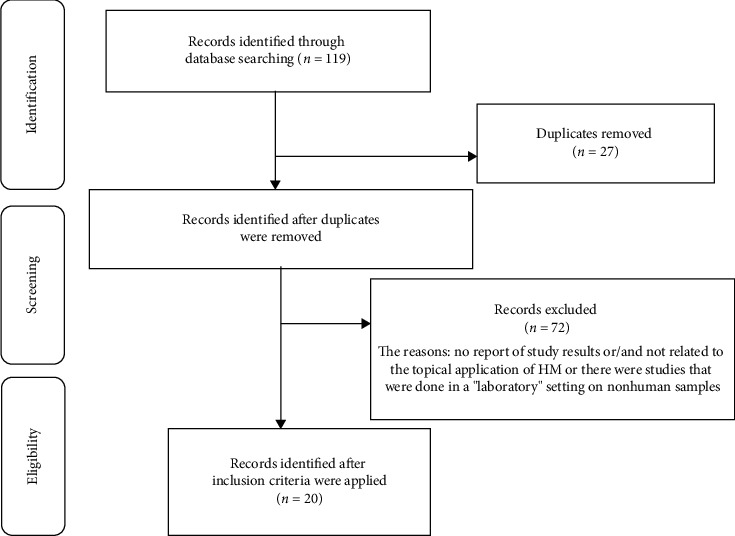
The articles included in the study.

Relevant articles were included through a three-step search strategy. Initially, 119 articles were obtained using the aforementioned keywords. These results were then screened using exclusionary criteria. A total of 27 articles were excluded as they were duplicated. For the rest of the articles, the exclusion criteria included (1) nonhuman samples and (2) protocol-based articles with no reported results. A total of 72 articles were excluded, leaving 20 articles for review (Figure 1).

### 2.4. Measurement

The author (ZSH) appraised each of the 20 articles which were peer-reviewed by another author (LAF) for accuracy. The extracted data included the title, country and city, participants characteristics, intervention description, control or/and comparison groups, length of follow-up, the measure of outcome variables, and main results (Table 1) (among the included studies, the results of which were reported in terms of hours, the results were rounded).

## 3. Results and Discussion

The HM has distinct anti-inflammatory components and properties that may explain the effectiveness of the HM when topically applied. These compounds include the following:

 
*Lipids*. The triacylglycerides, diacylglycerides, monoacylglycerides, free fatty acids, sphingomyelins, phospholipids, and cholesterol are the main lipids found in HM [32–34]. The HM lipids have been shown to provide a protective barrier against several types of invasive pathogens including *Group B Streptococcus* (GBS) on the mucosal surface [35].
 
*Proteins*. HM consists of over 400 various proteins including caseins, whey, and mucin which executes the inhibitory functions in response to pathogenic microbes [36]. Leucocytes as known anti-inflammatory components of the body floods account for 80%–90% of the HM proteins [9].
 
*Carbohydrates*. HM contains a vast range of complex carbohydrates. The most prevalent is lactose. Lactose is a disaccharide and consists of glucose which covalently bounds to galactose. Their function instead is to nourish the gastrointestinal microbiota [37].
 
*Antibodies*. The Secretory Immunoglobulin A (sIgA) in HM provides an immunological defense to protect the infant with the unmatured immune system against the pathogenic microorganisms [38]. There are also numerous antibody classes in HM which provide defense against neonatal GBS infection [39].

## 4. Topical Applications of HM (THM)

Almost all of the reviewed studies were focused on mothers and infants. The studies generally had consensus on the significant positive impacts of THM in women and their infants.

### 4.1. Effect of THM on Infants

The HM has been used in the bacterial colonization, umbilical cord separation time (UCST) [4], chlamydia conjunctivitis, rhinitis, ocular dryness, ocular lesions, diaper rash, eczema, and nipple sore [8] as well as bites, infectious wounds, burns, and rubbing [20].

#### 4.1.1. Umbilical Cord

An umbilical cord infection is the major cause of sepsis in newborns that may lead to death in some cases [16]. One of the major underlying causes of umbilical cord infections is a long separation time [40]. The WHO recommended keeping the cord dry as much as possible, and it also suggested a topical antiseptics such as chlorhexidine in poor health conditions and high levels of infections [41]. The THM is one of the effective traditional treatments commonly applied for shortening UCST or preventing and treating the umbilical cord inflammation in middle eastern.

In 2008, Taffazoli conducted a study to investigate the effect of THM on the bacterial colonization of the umbilical cord region. The findings revealed that first, *Staphylococcus epidermidis*, *Staphylococcus aureus*, *Escherichia coli*, and *Klebsiella pneumonia* were the most commonly grown organisms in the umbilical region; second, bacterial colonization was significantly lower in THM group compared with the control group (*P* < 0.001) [42].

In this regard, Ibhanesebhor and Otobo showed that the susceptibility level of *Escherichia coli* to colostrum and THM was 57% and 28%, respectively. The *Staphylococcus aureus* was reported to be sensitive about 50% and 0% to colostrum and THM, respectively [43]. In this regard, Ramsey et al. conducted an experimental study on 229 host cells infected with *Chlamydia trachomatis* in a laboratory environment. The milk (colostrum and noncolostrum milk) samples were obtained from 13 lactating postpartum women and the samples were added to the infected cells. They observed that 11, 2, and 1 of the samples showed 85%, 75%, and 44% chlamydia growth inhibition started within less than 15 minutes. It was also noticed that colostrum is more effective than mature milk. In addition, they suggested that the used dose plays the main role in the effectiveness of THM [44].

Literature also concerned with the umbilical cord separation time using THM in comparison with the dry-care control group (DCC). Allam et al. studied the effect of THM on UCST and bacterial colonization recruiting 400 neonates that are equally allocated into THM and DCC groups. The results indicated that the umbilical cord of 80% of the neonates in the THM group was separated from the 3rd to 4th days, and the rest of the group (20%) lost their umbilical cords on the 5th to 6th days. The UCST was significantly lower in the THM group (4 ± 20 days) than that the DCC group (7 ± 10 days) (*P* < 0.001). Also, *Escherichia coli* and *Staphylococcus aureus* were 2% less in the THM group compared with the DCC group [17]. To answer the same research question, Aghamohammadi et al. recruited 130 newborns randomly allocated into two equal THM and DCC groups. According to the results, UCST was significantly longer in the DCC group (8 ± 2 days) than that in the HM group (6 ± 1 days) (*P* < 0.001) [18]. Additionally, Pujar et al. also supported the results of the previous studies enrolling 60 newborns. The findings of this study showed that UCST was significantly shorter in the THM group (5 days) when compared to that in the control group (9 days) (*P* < 0.05) [19]. Furthermore, Dhanawade and Amiri-Farahani et al. confirmed the previous findings showing a significantly lower UCST in THM ((5–6) ± 3 days) compared with DCC group ((7–9) ± 3 days) (*P* < 0.001) [20, 21].

Studies also compared the THM efficiency with commonly applied conventional medications including ethanol, povidone-iodine, ethyl alcohol, silver sulfadiazine, and chlorhexidine. On this point, Golshan and Hossein conducted a randomized clinical trial (RCT) to compare the effects of THM, DCC, and ethanol on UCST and omphalitis on a total of 300 neonates. The UCST was significantly shorter in the THM than those in the DCC (*P* = 0.0001) and ethanol groups (*P* = 0.003). No significant difference was reported in the frequency of omphalitis in the three groups [22]. The findings from another RCT performed by Mahrous et al. on 100 newborns confirmed the results of the previous study. The UCST was lower (4 ± 1 days) in THM than the ethanol group (8 ± 2 days) (*P* < 0.001) [23].

In 2006, Vural and Kisa examined the effect of THM compared with povidone-iodine and DCC on UCST among 150 healthy neonates. The UCST was shorter in THM (7 ± 2 days) and DCC (8 ± 3 days) than that in the povidone-iodine group (10 ± 3 days) (*P* < 0.05) [4]. In the same year, Ahmadpour-Kacho et al. compared the effect of THM with 96% ethyl alcohol and silver sulfadiazine on UCST among 312 newborns. The findings of this research reported that UCST was significantly different in THM (5 ± 2 days) than those in the ethyl alcohol (6 ± 2 days), silver sulfadiazine (10 ± 4 days), and control groups (7 ± 2 days) (*P* < 0.001) [24]. Furthermore, Abbaszadeh et al. compared UCST between THM and chlorhexidine groups. The UCST was significantly shorter in the THM group (7 ± 2 days) than that in the chlorhexidine group (13 ± 7 days) (*P* < 0.001) [25]. The bacterial colonization and UCST were compared in the THM, 4% chlorhexidine, and DCC groups in premature neonates. The UCST was reported in the THM, chlorhexidine, and DCC groups as 9 ± 2, 14 ± 3, and 11 ± 3 days, respectively. The levels of bacterial colonization in THM, chlorhexidine, and DCC groups were 22.9%, 71.4%, and 2.9% in the 5 ± 1 days after intervention, respectively (*P* < 0.001) [16].

UCST is a complex process, in which the nucleophilic polymorphocytes penetrate the area between the body and cord, induce digestion of the umbilical cord and ultimately separate these two segments [41, 45–47]. Moisture and bacterial concentration are the most important factors that barricade the UCST process [40]. It is suggested that HM optimizes and accelerates the aforementioned process using its distinguished components including proteins and antibodies [48, 49]. The antimicrobial proteins can inhibit the pathogenic microorganisms by making the environment unpleasant for the commensal flora, the pH, or bacterial substrates. Additionally, some of these antimicrobial proteins can kill a broad spectrum of bacteria by various mechanisms [50]. Of these proteins, the leukocytes found in HM, along with other immunologic factors, play a crucial role in the removal of the umbilical cord [35].

The secreted HM in the first days after the childbirth is called colostrum. The colostrum contains substantial amounts of the aforementioned components compared with the mature HM, which may explain the effectiveness of this component when compared with mature HM [22].

#### 4.1.2. Diaper Dermatitis (Contact Dermatitis)

Diaper dermatitis (DR) is a common source of inflammation in neonates [51], and its prevalence has been reported to be up to 50% [52]. DR in the long term can damage the skin seriously leading to secondary infections and skin ulcers [53]. The long-term exposure with urine and feces may break down the skin integrity due to the presence of lipase and protease enzymes in urine [54]. Moreover, the analysis of urea by bacteria existing in the stool may cause increased ammonia and subsequently enhanced skin pH, which in turn makes the skin more susceptible to infection in infants who wear the diaper [55]. Several studies assessed the effect of HM on DR treatment.

Seifi et al. conducted an RCT to assess the impact of THM on DR with recruiting 30 neonates aged 0–12 months. The daily application of THM on the skin indicated a significant difference between the THM and the control group on the third day (*P* = 0.004) [26]. On the other hand, Gozen et al. compared the THM and a barrier cream among 63 neonates in an RCT design. Although no significant difference was found between two groups in terms of recovery length of DD (*P* = 0.294), a significantly higher wound healing was found in the THM than the barrier cream group (*P* = 0.002) [27].

The finding from relevant studies suggested that the presence of a variety of vitamins and minerals in HM makes the skin soft and smooth and prevents dryness and fragility, thereby preventing the penetration of foreign microorganisms through the skin [56]. HM accomplishes this function by its natural lactic acid that artificially existed in many skin creams and lotions. Additionally, fatty acids of HM have hydrophobic properties preventing the skin penetration and damages caused by urea and enzymes in the stool and urine [9].

#### 4.1.3. Atopic Dermatitis (Eczema)

Literature has shown that breastfeeding, especially in the first weeks of postpartum, has a significant influence on reducing allergic disorders including atopic dermatitis (AD) [4]. Some studies have shown that the application of HM topically can also heal the AD.

Kasrae et al. showed that THM can be effective in shortening the AD duration as much as hydrocortisone (1%) in children [13]. However, the findings from the study conducted by Berents et al. comparing the THM and control groups did not show a significant difference between the two groups [28].

HM consists of many natural immunologic and anti-infective agents that have strong antimicrobial functions, which offer specific and nonspecific passive immunities [57]. Apart from that, the fatty acids including *n*‐3 long‐chain polyunsaturated fatty acids (LCPs) and ruminant fatty acids have an important role in the curative effect of HM [58]. Also, a group of inflammatory cytokines such as IL-I*β* as well as CD14 protein are shown to be associated with eczema [59].

#### 4.1.4. Eye Disease

The use of HM in the treatment of eye diseases was investigated in a study by Gagnon who lived in a marginalized area of Malawi. Due to the lack of access to the required treatment facilities, the researcher tended to use traditional methods, such as THM in the treatment of optic inflammation [60]. Also, a retrospective study assessed the effect of THM on the treatment of congenital nasolacrimal duct obstruction (CNLO) in children. The evidence revealed that the treatment with local antibiotics took a significantly higher time (5 days) than that in the THM group (1 day) (*P* < 0.001) [29]. Furthermore, Ghaemi et al. examined the prophylactic effect of colostrum on neonatal conjunctivitis. In this study, a total of 268 neonates were allocated into three groups: colostrum (*n* = 89), control (no intervention) (*n* = 97), and topical erythromycin (0.5%) (*n* = 82) groups. According to results, the frequency of conjunctivitis was higher in the control group, in comparison with those receiving topical colostrum and topical erythromycin (0.5%) (*P* = 0.03) [30]. A similar study in 1998 on 565 newborns had demonstrated that conjunctivitis occurred in 9.1% and 25.6% of the children in the HM and control (no intervention) groups, respectively, showing a significant difference between two groups (*P* < 0.00001) [31].

The explored promising effects of HM and especially colostrum on optical diseases may be due to the presence of a great number of antibacterial agents [38]. The lipids in HM seem to mediate the association between THM and conjunctivitis by protecting the body against invasive infections [35].

### 4.2. Effects of HM on Mothers

Multiple studies are concerned with THM effects on a variety of skin damages and infections in mothers. These include the following:

 
*Nipple Sore.* Nipple irritation is one of the most common complications in breastfeeding women. It was reported that 96% of mothers tend to not lactate due to nipple pain and ulcer during breastfeeding [61]. Since nipple-related damages and subsequent pains are important factors in the mother's decision to stop lactation, choosing the appropriate intervention is a dire need. Mohammadzadeh et al. assessed the effect of HM and lanolin on the sore nipple. The data from the study demonstrated a longer recovery time in the lanolin group when compared to those in the THM group (*P* = 0.029) [8].
 
*Perineal Ulcer.* Approximately 85% of women undergo the episiotomy (perineal area cut) during labor, of which 69% need suture in the area [62]. Due to the high moisture and low ventilation, the perineal area seems to be a proper environment for the growth of bacteria and invasive microorganisms [7, 63]. The perineal inflammations lead to the mother's pain and discomfort and consequently reduced the ability to take care of herself, the baby, and the family. Studies also have indicated the septic shock and death in long-term infection of the perineum [64].

Admasari et al. suggested THM as an alternative method to the perineal ulcer. Their study aimed to compare the healing of the perineal ulcer in the povidone-iodine (10%) and THM groups. The results showed that the THM group (11 days) had a significantly shorter wound healing process than the povidone-iodine (10%) (20 days) groups (*P* = 0.002) [7]. The stem cells in the HM, along with other anti-inflammatory and antioxidant components, may explain the effectiveness and noninvasive protective properties of HM against various types of ulcers [65].

The usage of HM for numerous types of wounds has been known for several decades in some countries [17]. Some of the beneficial effects of HM in response to life-threatening inflammations on mother and infant may be related to the biologically active factors including growth factors such as epidermal growth factor (EGF) and insulin-like growth factor (IGF) superfamily and CD14 protein as well as adipokines and inflammatory cytokines [59].

Growth factors are necessary elements to activate the process of granulation (inflammatory phase) and epithelization (proliferative phase), as required phases for wound healing [66]. IGF-1 as a unique independent growth factor attributes an anabolic feature which helps alleviate skin or mucosal injuries and also diminish the catabolism process [4].

Also, a group of bioactive and anti-inflammatory molecules were known as proresolving mediators (SPMs) that can eliminate the infectious microorganisms, inflammation, and pain in the wound area. Even though the exact mechanism of action remains uncertain, it seems that HM may be a safe and natural substance against foreign and invasive microorganisms due to its rich source of growth factor, anti-inflammatory agents, antibacterial agents, and immunity factors [7].

### 4.3. Limitations and Future Research

The reviewed research articles displayed the positive effect of THM on various damages such as dermal and mucosal injuries. However, more research with a homogenous methodology is needed to confirm the potential advantages and disadvantages of THM on the discussed injuries. Additionally, the examination of THM on the different damages and pathogenesis may be beneficial to reach certainty in terms of curative properties of THM. In this review paper, we only gathered recent and basic articles to help shed light on the topical effects of HM. Therefore, other articles on this topic may exist that were outside of our specified date range.

## 5. Conclusion

According to the results of the current set of studies reviewed, there is a general agreement on the positive effects of the THM on the prevention and treatment of common maternal and infantile complications including lesions and sores with/without secondary infections. To date, no adverse side effects following the THM application have been reported. HM may be an accessible, safe, and appropriate alternative for the treatment of damages of skin and mucus tissues with various origins in mothers and infants.

## Figures and Tables

**Table 1:** Randomized trials evaluating the impact of topical application of HM on maternal and child health outcomes.

Author, year, and location	Study groups	Intervention	Variable measured/scale	Results
*Effect of THM on infants*				
*Umbilical cord*				
Lyngdoh et al., 2018, India [16]	HM (*n* = 35); chlorhexidine (*n* = 35); DCC (*n* = 35)	HM and chlorhexidine: topical application of HM or 4% chlorhexidine was done by sterile swab once a day till the cords fall off; DCC: NI.	UCST	HM: 9 ± 2; chlorhexidine: 13 ± 3; DCC: 11 ± 3 days (*P* = 0.001)
Allam et al., 2015, Egypt [17]	HM (*n* = 200); DCC (*n* = 200)	HM: mothers used about 4–6 foremilk (before lactation) cord stump and let the milk to completely dry. The starting time for applying the pertinent substances is considered 4 hours after birth. The researchers asked the mother to apply milk drops 3 times a day since the 4th hours of birth until UCS and 2 days after; DCC: NI.	UCST	HM: 4 ± 20; DCC: 7 ± 10 days (*P* < 0.001)
Aghamohammadi et al., 2012, Iran [18]	HM (*n* = 65); DCC (*n* = 65)	HM: mother's secreted milk was directly applied to the remaining part of the cord and allowed the milk to get completely dry. This intervention beginning time was 3 hours after birth and then every 8 hours for 2 days after UCST; DCC: NI.	UCST	HM: 6 ± 1; DCC: 8 ± 2 days (*P* < 0.001)
Pujar et al., 2013, India [19]	HM (*n* = 30); DCC (*n* = 30)	HM: fresh mother milk was applied using a sterile swab twice in a day for 3 days; DCC: NI.	UCST	HM: 5; DCC: 9 days (*P* < 0.05)
Dhanawade, 2014, India [20]	HM (*n* = 45); DCC (*n* = 45)	HM: approximately 1 ml of fresh mother milk is applied by using sterile buds on the cord stump and let them 10 minutes dry; DCC: NI.	UCST	HM: 5; DCC: 9 days (*P* < 0.05)
Amiri-farahani et al., 2007, Iran [21]	HM (*n* = 61); DCC (*n* = 57)	HM: mothers applied their foremilk to their infants' umbilical stump by 3 h after birth and continued every 12 h until 2 days after UCS; DCC: NI.	UCST	HM: 6 ± 2; DCC: 7 ± 3 days (*P* < 0.016)
Golshan and Hossein, 2013, Iran [22]	HM (*n* = 100); DCC (*n* = 100); 70% ethanol (*n* = 100)	HM: mothers in groups HM and ethanol washed the area of the umbilical cord with their milk and ethanol 70%, respectively, with the frequency of twice a day for two days after UCS; DCC: NI.	UCST	HM: 7 ± 2; DCC: 8 ± 2; ethanol: 9 ± 2 days; the results showed significant difference of UCST between HM and ethanol (*P* < 0.0001) and DCC groups (*P* < 0.003)
Mahrous et al., 2012, Egypt [23]	HM (*n* = 50); 70% ethanol (*n* = 50)	HM or 70% ethanol: mothers of each group applied pertinent substances topically every 8 hours to the area until two days after USC and allow them to dry without washing them out.	UCST	HM: 4 ± 1; 70% ethanol: 8 ± 2 days (*P* < 0.001)
Vural and Kisa, 2006, Turkey [4]	HM (*n* = 50); DCC (*n* = 50); povidone-iodine (*n* = 50)	HM: fresh mother milk was directly applied to the distal end edge of the stump twice a day for 2 days after the cord fell off; DCC: NI; povidone-iodine group: they used povidone-iodine to completely cover the cut edge of the stump twice a day umbilicus for 2 days after UCS.	UCST	HM: 7 ± 2; DCC: 8 ± 3; povidone-iodine: 10 ± 3 days (*P* < 0.05)
Ahmadpour-Kacho et al., 2006, Iran [24]	HM (*n* = 79); DCC (*n* = 78); silver sulfadiazine (*n* = 77); ethyl alcohol (*n* = 78)	HM, silver sulfadiazine, and ethyl alcohol groups received topical application of respective substances by a sterile gauze or swab on umbilical stump area 3 hours after birth time and continued every 8 hours till two days after UCS; DCC: NI.	UCST	Ahmadpour-Kacho et al., 2006, Iran [24]
Abbaszadeh et al., 2016, Iran [25]	HM (*n* = 80); chlorhexidine (*n* = 82)	HM (fresh milk) and chlorhexidine groups received topical application of pertinent material to the cord region from three hours after birth and every 12 hours till 2 days after the UCS.	UCST	Abbaszadeh et al., 2016, Iran [25]

*Diaper dermatitis (contact dermatitis)*				
Seifi et al., 2017, Iran [26]	HM (*n* = 15); C (*n* = 15)	HM: they applied HM 3 times a day on the diaper area and allowed the area to dry before changing nappies; C: NI.	Diaper dermatitis (DR) severity/five-point scale	HM: 0 ± 0; C: 1 ± 1 (*P* < 0.05)
Gozen et al., 2014, Turkey [27]	HM (*n* = 30); barrier cream (zinc oxide 40%; cod liver oil) (*n* = 33)	In both groups of HM and barrier cream, pertinent topical applications were applied to the diaper area during each diaper change for maximum 5 days. Diapers were changed every three hours (eight times a day).	Clinical improvement/four-point scale	HM: 60%; barrier cream: 93% (*P* < 0.002). HM: 4 ± 1; barrier cream: 4 ± 1 days (*P* = 0.27)

*Atopic dermatitis (eczema)*				
Kasrae et al., 2015, Iran [13]	HM (*n* = 54); 1% hydrocortisone ointment (*n* = 50)	HM: mothers were recommended to rub the hindmilk twice a day on the affected area; 1% hydrocortisone ointment: a thin layer of ointment was applied twice a day to all areas of the actively diseased skin.	Atopic dermatitis (AD) severity/SCORAD	HM: 1 ± 3; 1% hydrocortisone ointment: 2 ± 3 (*P* = 0.43)
Berents et al., 2015, Norway [28]	HM (*n* = 9); emollient (*n* = 9)	HM: by hand milking, the mothers were to squeeze out and throw away the first few droplets of milk and then squeeze the next droplets directly from the nipple to the eczema spot. HM and emollient: both groups were treated with moisturizing cream and this regimen continued three times a day for four weeks.	Severity of atopic dermatitis/SCORAD	No effect was found on eczema spots treated with topical application of fresh HM

*Eye diseases*				
Verd, 2006, Spain [29]	HM (*n* = 45); antibiotic eye drops (*n* = 20)	HM or antibiotic eye drops: the mothers applied HM or antibiotic eye drops by physician prescription prospectively.	CNLO/duration of treatment	HM: 1; local antibiotic: 5 months (*P* < 0.001)
Ghaemi et al., 2014, Iran [30]	Colostrum (*n* = 89); topical antibiotic (*n* = 82); C (*n* = 97)	Colostrum: they disinfected the nipple first and then used a 10 cc sterile syringe with a sterile bistoury for sucking the colostrum and then applied 2 drops of it in each eye; topical antibiotic: they treated with topical erythromycin ointment (0.5%); C: NI.	Clinical conjunctivitis/eye discharge culture for 28 days	The frequency of conjunctivitis in colostrum was higher than the topical antibiotic group and lower than the control group (*P* = 0.03)
Pishva et al., 1998, Iran [31]	HM (*n* = 327); C (*n* = 238)	HM: requested to instill one drop of their HM into each eye of the baby prior to each breastfeeding (at least four times a day) for the first ten days; C: NI.	Neonatal conjunctivitis occurrence/prophylactic effects	HM: 9.1%; C: 25.6% (*P* < 0.0001)

*Effects of HM on mother*				
Mohammadzadeh et al., 2005, Iran [8]	HM (*n* = 78); lanolin (*n* = 74); C (*n* = 73)	HM: mothers rubbed hind milk on the sore area after each breastfeeding; lanolin: mothers were asked to use lanolin locally on the sore and clean it before infant feeding; C: NI.	Healing length by observation and questionnaire on the 3rd, 5th, 7th, and 10th days	The healing time in the lanolin group was longer than the HM (*P* = 0.029) and the C group (*P* = 0.028)
Admasari et al., 2017, Indonesia [7]	HM (*n* = 15); C (*n* = 15)	HM: topical application of fresh mother milk; C: povidone-iodine (10%). In both groups, pertinent substances were applied to the wound area with a cotton tissue twice a day.	Perineal ulcer severity/REEDA	HM: 11; C: 20 (*P* value < 0.05)

AE: atopic eczema; C: control; CNLO: congenital nasolacrimal duct obstruction; DCC: dry cord care; GA: gestational age; HM: human milk; NICU: neonatal intensive care unit; NI: no intervention; UCS: umbilical cord separation; UCST: umbilical cord separation time.

## Abbreviations

WHO: World Health Organization
CINAHL: Cumulative Index to Nursing and Allied Health Literature
GBS: Group B Streptococcus
UCST: Umbilical cord separation time
AE: Atopic eczema
SCORAD: SCORing Atopic Dermatitis
LCPs: Long‐chain polyunsaturated fatty acids
TGF-A and TGF-B: Transforming growth factors of alpha and beta
IGF-1 and IGF-2: Insulin-like growth factors 1 and 2
HM: Human milk
THM: Topical human milk
DCC: Dry cord care
NICU: Neonatal intensive care unit
UCS: Umbilical cord separation
NI: No intervention
GA: Gestational age
AE: Atopic eczema
C: Control
CNLO: Congenital nasolacrimal duct obstruction.
